# A scanning electron microscopy study of projectile entry fractures in cortical bone; genesis and microarchitectural features

**DOI:** 10.1007/s00414-021-02743-4

**Published:** 2021-12-13

**Authors:** John M. Rickman, Jonathan Painter, Rachael Hazael

**Affiliations:** Defence Academy of the United Kingdom, Cranfield Forensic Institute, Cranfield University, Shrivenham, SN6 8LA UK

**Keywords:** Forensic anthropology, Projectile trauma, Ring cracking, Cone cracking, Plastic deformation, Shock waves, Hydroxyapatite crystals

## Abstract

The present paper presents a scanning electron microscope (SEM) analysis of the genesis and microarchitecture of experimentally induced cortical entry fractures in porcine scapulae impacted at velocities ranging from 54 to 897 m/s. SEM observation was conducted on polyurethane replicas cast from negative silicone moulds. Analysis of the sequence of fracture processes operative during projectile impact revealed the presence of ring cracks at the site of impact, confirming that penetration in sandwich bones is achieved by cone crack propagation. Despite impulsive loading, two forms of plastic deformation were identified in the cortical bone surrounding the entry fracture up to a maximum velocity of 871 m/s. Microscopic radial and concentric cracks were associated with projectile impact, and the role of pores and pits as stress concentrators was captured. Possible underlying mechanisms for the observed plastic deformation are described, and the diagnostic utility of SEM analysis is presented.

## Introduction

**Fig. 1:**
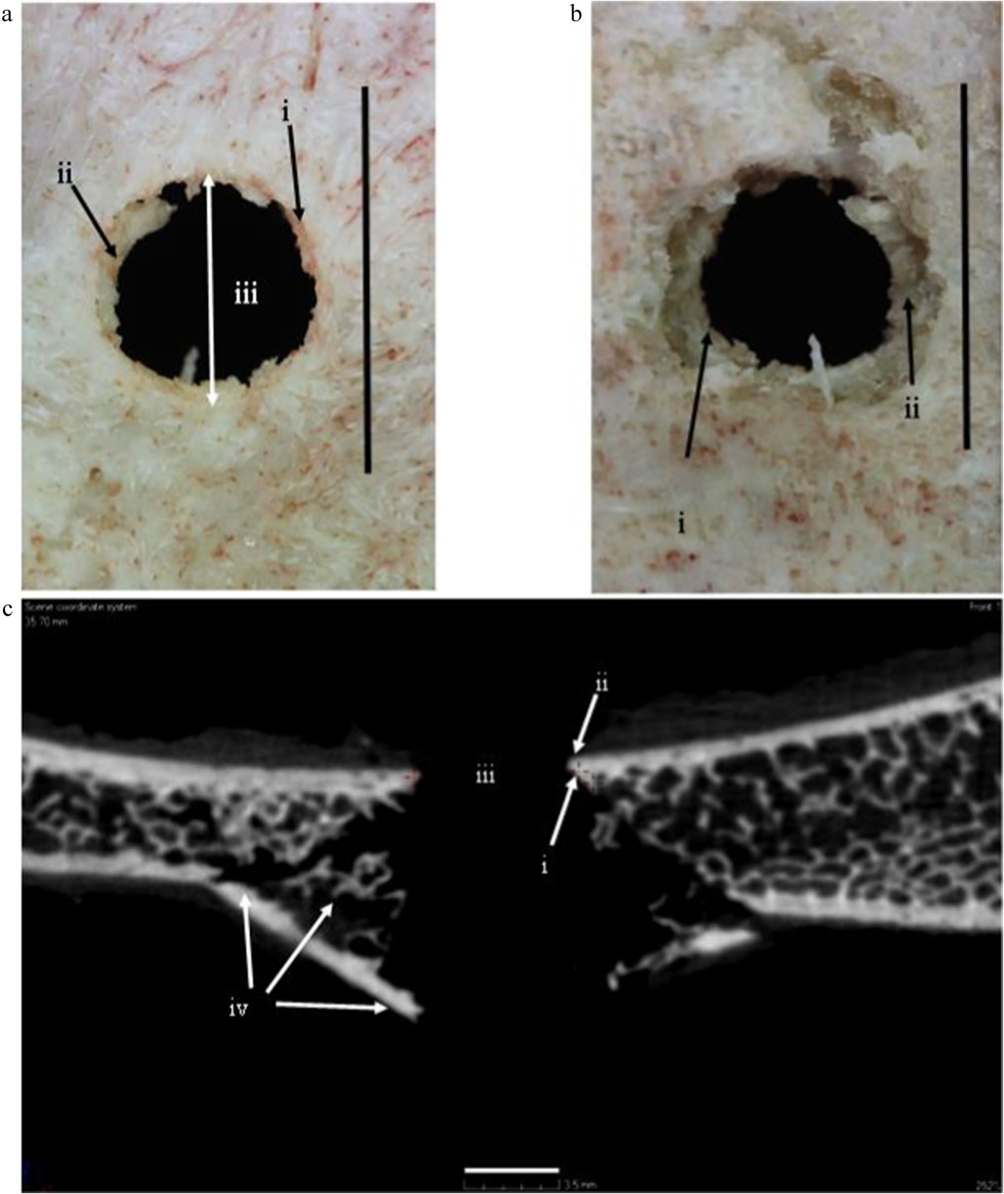
Key nomenclature for the components of a bevelled fracture in sandwich bones; **a–b**, 163 m/s perforating impact (*Ei* 11.78 J, E´ 0.42 J/mm^2^; scales bars 10 mm); **a**, **i**, apex of the entry cortical fracture edge (EnCF); the top edge of EnCF where the cortical fracture edge and cortical surface meet; the EnCF itself runs under the apex and is not visible from above; **ii**, cortical roof fragment; fragmentary component of the conoid roof remaining in situ after fragmentation and ejection of the bulk of the conoidal volume; the distal edge faces the cortical entry and the medial edge the cortical surface; **iii**, cortical entry; delineated in cross section by the EnCF and in surface view by the apex of the EnCF; **b**, same cortical entry as in seen in **a** but from the bevel side; **i**, EnCF angling away from the apex; **ii**, underside of the cortical roof fragment seen in **a**; **c**, 158 m/s perforating impact (*Ei* 11.05 J, E´0.39), µCT cross-sectional view showing internal structure; (scale bar 3.5 mm) i, EnCF, ii, apex of the EnCF, iii, cortical entry; iv, fragment of conoid

The typical fracture produced by perpendicular projectile impact in tri-layered sandwich bones exhibits a circular hole in the outer cortical layer (the *cortical entry*) and a bevel flaring in the direction of projectile travel on the exiting side [1, 2]. This unique fracture type is composed of distinct morphological sub-components which for the purposes of discussion are identified and defined in Fig. 1 [3]. Recent work indicates that conoidal fractures are produced by propagation of a Hertzian cone crack through the three laminae of the sandwich bone [3, 4]. This translaminar fracture mechanism results in the production of tri-layered conoids of bone that typically undergo comminution during high-velocity impact, although traces may remain in the form of cortical roof and floor fragments (Fig. 1). The correlation between this distinct conoidal fracture morphology and bullet involvement led to it being considered diagnostic of ballistic impact quite early in skeletal trauma analyses [2], and it is largely still considered so today [5]. However, recent case reports of conoidal fractures resulting from low-velocity impacts that did not involve bullets, discussed below, have partially reduced the diagnostic utility of this fracture morphology in identifying gunshot trauma. In addition, in skeletonised and/or fragmentary material, multiple factors can add considerable complexity to the differential diagnoses of perforating holes in skeletal elements [6].

In the first instance, potential sites of gunshot trauma in skeletonised remains must be differentially diagnosed from holes arising from congenital abnormalities, disease and surgical alteration [7]. Taphonomic inputs can further muddy the diagnostic waters by masking or mimicking gunshot trauma. For example, animal trophic activities may damage fracture margins [8], whilst exposure to fire may produce bevelled defects resembling conoidal wounds [9, 10]. Differential diagnosis may be challenging in thinner cranial bones, such as the sphenoid, which do not exhibit bevelling [6]. In such cases, a gunshot wound may be identical to holes created by other mechanisms such as the punctures created by mammalian [11] and avian scavengers [12]. Finally, fragmentation may make diagnosis difficult if only a small portion of a penetrating defect is recovered [13–15].

The cone cracking mechanism underlying bevel formation does not require projectile perforation and is also independent of impact velocity [3, 4]. This common fracture mechanism across loading rates explains why bevelling has been described subsequent to lower velocity impacts with a range of implements including a bike lock [16], umbrella tip [17] and umbrella vane [5], and dictates that the cortical entry/bevel combination is not diagnostic of high-velocity trauma. The diagnostic value of radial and concentric cracks is limited by the fact they are not always present with ballistic trauma; accordingly, their absence cannot rule out bullet involvement [5]. This diagnostic complexity, coupled with a deficit in diagnostic techniques, means that current practice is to assume all traumatic bevelled perforations are gunshot wounds in the absence of evidence to the contrary [5]. Overlap of this magnitude between trauma types is readily explained by the biomechanical continuum, a concept which considers a given traumatic fracture to be the product not just of weapon type but of multiple interacting factors such as the applied force, the surface area of the impacting object and the rate of acceleration or deceleration [18].

The biomechanical continuum is an important concept because it highlights that sharp, blunt and projectile trauma are not absolute categories into which all traumatic features can or should be placed [19]. However, these traditional categories remain critical to law enforcement agencies due to their role in eliminating or reducing the number of potential weapons underlying a given traumatic feature [20]. It is therefore essential that further research be undertaken to investigate techniques that may be applied to enhance differential diagnosis when bones expressing a hole of ambiguous aetiology are recovered. Such techniques may be derived from one of two categories:

1. The detection of trace evidence associated with firearm discharge (gunshot residue, GSR) and/or material transferred during bullet contact with soft tissue or bone (bullet wipe).
2. Analysis of bone trauma morphology including fracture characteristics, cortical surface features (topography) or other alterations in bone structure or organisation.

To date, the majority of investigations have been conducted in the first category, and the detection of GSR on bone has proven to be quite a powerful diagnostic technique [13–15, 21]. However, the presence of trace signatures on bone is dependent on projectile type [22], firing distance [23] and taphonomic exposure [13, 21], and an absence of GSR does not rule out bullet involvement. The exacting requirements of proof in legal settings also dictate that GSR is of no use if environmental contamination cannot be ruled out [15].

At present there is no macroscopic method to accurately determine projectile velocity from bone trauma morphology, in large part due to the multiple intrinsic and extrinsic factors that determine the nature and extent of fracture [24]. However, when considering the utility of alteration in bone morphology as a diagnostic indicator, it is important to note that bone is a complex hierarchical material with seven levels of organisation spanning from the nanoscale (hydroxyapatite crystals and collagen fibres) to the macroscale (the whole bone) [25]. Whilst analysis at the macroscale can reveal important information about skeletal trauma, it cannot, in isolation, detect alterations at microstructural scales. In the first instance, fracture propagation does not just occur through the macrostructural features of bone; rather, the crack tip propagates through the microstructural features of bone. Moreover, high-velocity projectile impact exposes bone to a unique set of conditions characterised by extreme stresses acting in short time spans [26], shock-wave propagation [27, 28] and frictional heating [28]. Considering these intense conditions, it is reasonable to ask if there might be a scale at which high-velocity impact results in detectable changes in bone structure and/or morphology that would enable enhanced differential diagnosis of gunshot trauma.

At present, our understanding of the microstructural features of cortical entry fractures is limited, and the analyses that have been conducted have all utilised a narrow range of impact velocities. Scanning electron microscopy (SEM) formed a prominent component of all these studies due to its great depth of field, high resolution and large magnifying power which make it eminently suitable for surface analysis. Speeter and Ohnsorge [29] impacted 49 human femora and found that shot direction was recorded at the microscopic scale in both cortical and trabecular bone. Significantly, these authors also reported striated abrasions resembling bullet rifling marks in the cortical bone. Gaîdash et al. [30] utilised SEM and atomic force microscopy (AFM), in addition to spectroscopic and nanohardness measurements, to analyse cortical entry fractures in human bones. These authors reported an increase in porosity of the cortical bone, which they termed shock wave osteoporosis, and a decrease in bone hardness towards the perforated region which they attributed to shock wave-induced changes in the mineral phase of the bone matrix. More recently, Keiser et al. [31] impacted pig ribs with 0.22 handgun bullets and analysed the wound perimeter with SEM. These authors described areas of smoothed cortical bone on the entry cortical fracture edge (EnCF) which they attributed to frictional melting of the hydroxyapatite.

Rickman and Smith [6] utilised SEM to analyse experimentally induced conoidal wounds in bovine scapulae with two types of rifle ammunition (soft point and full metal jacket, FMJ) and a captive bolt gun. Large microcracks were observed emanating from the EnCF in both soft point and FMJ wounds, and entry-exit and side to side directionality were recorded in deviations of microscopic bone fragments. Although the loading rates experienced during high-velocity impacts should result in brittle fracture [32], a notable morphological feature produced by the FMJ projectiles and captive bolt impacts was a conspicuous plastic deformation of sections of the perimeter of the cortical entry in the direction of projectile travel. The mechanism or mechanisms underlying this paradoxical plastic deformation currently remain unknown, as does the full extent of its distribution around a single cortical entry.

In order to examine the micromorphology of cortical entry fractures and to identify features that might be of potential diagnostic utility, the present paper presents the findings of an SEM analysis of projectile fractures induced in the cortical bone of porcine sandwich bones at impact velocities ranging from 54 m per second (m/s) to 897 m/s. Since the fracture mechanisms operative during cortical entry formation may themselves be of diagnostic utility, the sequence of fracture events occurring during penetration and perforation is presented. This is followed by an analysis of the effects of projectile impact on cortical bone at and adjacent to the cortical entry. The significance of these findings in relation to differential diagnosis is then discussed.

## Methods

### Sample, shooting equipment and specimen preparation

The specimens utilised for SEM analysis formed part of a previous experimental series that was subjected to microcomputerised tomographic (µCT) and videographic analyses; detailed descriptions of methodology can therefore be found elsewhere [3, 4]. Adult (12–14 months) porcine (*Sus scrofa domesticus*) scapulae were selected for impact due to their sandwich construction and the fact they express conoidal fractures when impacted by projectiles. Histologically, artiodactyl bone is largely of fibrolamellar organisation and thus differs from the secondary osteons found in adult human bone. However, the laminated morphology of fibrolamellar bone is broadly comparable to the layered, fibrous sheets of bone that form the superficial layer of cranial vault bones [33]. Scapulae were sourced from animals humanely killed as part of the food chain. The infraspinous fossa was chosen for the target area due to its larger size, and only specimens with a covering of soft tissue in this fossa were selected. Each scapula was machined on a bandsaw to provide relatively uniform plates for impact. Specimen size after cutting was determined by scapula dimensions and robusticity but was typically 12 cm high by 13 cm wide; all specimens were large enough to ensure the selected impact point was a minimum of 3 cm from an edge to eliminate shock-induced edge effects. Specimens were stored frozen prior to impact and defrosted the day of the experiment.

**Fig. 2:**
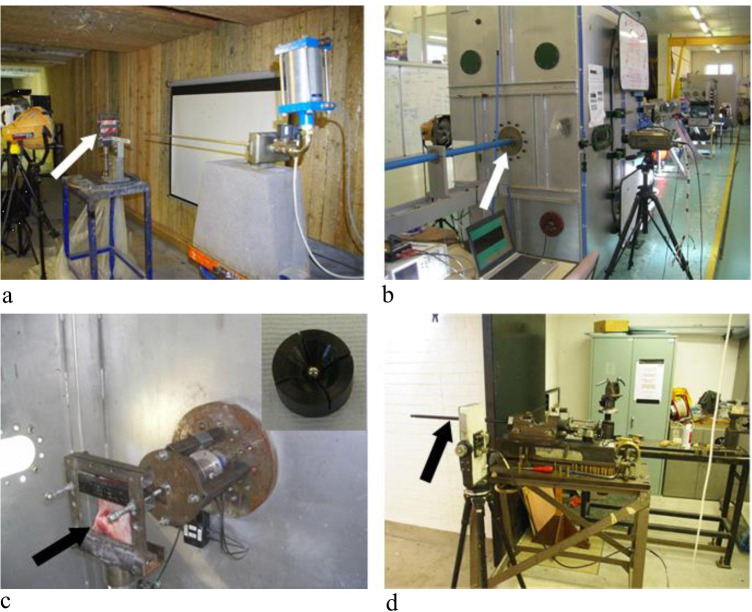
Projectile delivery systems; **a**, compressed air gun with 6 mm barrel situated in front of a clamped specimen (arrow); **b**, 21.3 mm calibre gas gun chamber with high-speed camera located on a tripod at the viewing window; the 21.3 mm barrel enters the chamber at left (arrow); **c**, view inside the 21.3 mm gas gun chamber showing specimen clamped in front of the barrel (arrow); part of the viewing window is illuminated at left; inset shows a 6 mm projectile housed in a plastic sabot; **d**, image of Enfield number three proof housing with barrel located at left (arrow)

To eliminate effects of projectile shape and deformation on perforation mechanisms, 6-mm surface hardened carbon steel spheres were utilised (Atlas Bearings LTD, UK). This approach was selected as it allows the possible effects of other variables, such as projectile deformation, to be isolated and characterised in future experimental analyses. A compressed air gun with 6-mm barrel was utilised to impact specimens from 54 to 96 m/s (Fig. 2a). Pressure values required for a given velocity were established using test shots to create a calibration curve of pressure versus velocity. Velocities of 149–333 m/s were achieved with a 21.3 mm calibre gas gun using sabot-mounted projectiles (Fig. 2, b–c). In this system, specimens were impacted inside a gas gun chamber; a viewing window on one side allowed horizontal positioning of a Phantom high-speed camera (Fig. 2b). Inside the chamber, specimens were held in a metal clamp in line with the viewing window (Fig. 2c); a light shone down the barrel allowed the target area for impact to be selected. Plastic sabots were fragmented by a sabot stripper located at the exit of the barrel; this process was assisted by horizontal machined cuts made across the sabots (Fig. 2c, inset). Incident velocities from 591 to 897 m/s were achieved using an Enfield number three proof housing with 7.62-mm barrel (Fig. 2d), again using sabot mounted projectiles. The sabots were situated inside a cartridge which was then filled with the appropriate amount of propellant to achieve a given velocity using a standardised chart for use with the proof housing.

Unless filming from the front or rear, impacts were filmed from the side with a scale for determining incident (pre-impact) and residual (post-impact) velocities. In the 21.3-mm gas gun, three specimens were filmed from either the front or rear to capture the entry and exit process (see [3]). It was still possible to derive an estimate of incident velocity in these specimens using a time gate fitted to the gas gun, which measured velocity as the projectile passed through two infrared beams (estimated velocities 165 m/s, 158 m/s and 333 m/s, respectively). When front and/or rear footage was obtained using the air-powered gas gun (two defleshed re-bound specimens), incident velocity was estimated from the calibration curve (~ 57 m/s for both specimens). In the absence of direct measurement of incident velocity, kinetic energy data therefore was not calculated for these specimens. Individual velocities (m/s) for each filmed impact were calculated as the mean of three repeat measurements in Phantom Cine viewer software. Pre-impact (incident) kinetic energy (*E*_*i*_) in joules (J) for each impact was calculated from the mean incident velocity according to the formula:

1 $$\frac{m{v}^{2}}{2}$$

where *m* is the mass of the projectile in grammes and *v* is the velocity of the projectile in m/s. The amount of kinetic energy per unit cross-sectional surface area of the projectiles (E´, kinetic energy density, J/mm^2^) was calculated using the following formula:

2 $$\frac{{E}_{i}}{A}$$

where *E*_*i*_ is the incident kinetic energy and *A* is the cross-sectional surface area of the projectiles, $(\pi {r}^{2}$= 28.27 mm^2^). Incident velocity, *E*_*i*_ and E´ are provided in the figure legends for each micrograph.

Subsequent to impact, specimens were photographed and re-frozen to prevent soft tissue degradation whilst specimens awaited analyses. Previous µCT comparison of pre- and post-freeze damage architecture in control specimens revealed no difference in fracture patterns after freezing [3]. For SEM analysis, specimens were defleshed by cold water maceration in open plastic tubs under a fume hood. Bulk soft tissue was carefully removed prior to immersion to leave as little as possible for the maceration period. Specimens were photographed prior to an immersion of 7 days. After this period, soft tissue was carefully removed using a scalpel, metal scraper, and plastic and wood implements; only the latter two were used in closer proximity to the cortical entry. Great care was taken to avoid tool marks on the bone by avoiding their use in an area of approximately 1–1.5 cm around the cortical entry. After cleaning, specimens were photographed, air dried for 7 days, and then re-frozen to prevent further degradation of the organic and mineral phases, whilst other specimens underwent maceration. In total 26 specimens were utilised for analysis; 22 of these were perforated specimens, whilst four were depressed factures caused by re-bound impact (two impacted fleshed and two impacted defleshed for filming). One perforated specimen was utilised as a test bone to perfect the replication technique.

### SEM negative moulding and positive casting

All machined specimens were too large to fit in the SEM chamber, and it was therefore necessary to make replicas of the cortical entries for analysis in the SEM. Replication was achieved using Isomark T-2 grey silicone impression material (Isomark Ltd., Leicestershire, UK). T-2 was selected as its longer working life (3 min at 20 °C) allowed multiple specimens to be replicated before the silicone cured in the applicator nozzle. Silicone was applied using the 50-ml cartridge system and applicator gun with attached mixing nozzle.

In order to cast both the EnCF and surrounding cortical surface, it was necessary to form a floor to the cortical entry. Testing revealed that the most cost-effective and simple material for this purpose was adhesive wall putty, which is readily available from stationary suppliers. Loose cortical floor fragments that obstructed cortical entry access via the bevel were removed where possible. A portion of putty was rolled into a ball and one end then rolled tighter until it was just slightly larger than the cortical entry. A round-ended clay sculpting tool was then used to form a slight indentation in the end of the elongated portion. The putty was then introduced through the bevel and, viewing from the cortical entry side, pushed into place such that the edges of the putty indentation approximated the lower aspect of the EnCF. Larger gaps between putty and EnCF, which sometimes formed due to irregularity in bone morphology on the bevel side, were removed as much as possible by very carefully placing a round-ended sculpting tool through the cortical entry and gently nudging the putty to reduce the size of the gap. At all times, great care was taken not to touch the EnCF with the tool or putty.

Specimens were subsequently placed on a flat surface, and the T-2 introduced as close to the putty floor as possible; enough was added to create a raised protrusion of T-2 approximately 5 mm or more above the cortical entry. A square section of clear 0.5-mm thick plastic measuring 15 mm × 15 mm was then used as a cover slip to gently press down on the T-2. This gentle pressure ensured the impression material spread over the cortical surface and also provided a flat back to the negative mould. To ensure that curing had occurred, a small quantity of T-2 was placed on the cortical surface of the specimen without a cover slip, thus enabling its hardness to be felt. The mould was photographed in position, and, when cured, the top was marked with a coloured dot. When removing the moulds, the putty was detached first to prevent pinching of the negative mould against the bone; the silicone moulds were then carefully removed and placed in small re-sealable plastic containers.

Negative silicone moulds were cast to positive replicas using Easy Flo 60, a two part (A and B) cold curing polyurethane resin (Polytek Development Corp., Pennsylvania). Positive replicas were cast in circular pots with a removable floor. Negative moulds were stuck to the removable pot base using double-sided sticky tape; to maintain specimen orientation, the top of the replicated entry fracture was marked on the outside of the pot with a sticky label. Equal parts A and B were mixed and gently stirred for 1 min before pouring into the pots; the resin was then allowed to cure for 90 min. To enable orientation in the SEM, the top of the cortical entry was marked on the reverse side of the replica after curing. Positive replicas were then stored in individual plastic containers and left to fully cure for 7 days before analysis. Replicas were analysed in a Hitachi SU 3500 SEM in variable pressure mode (80 Pa) with an acceleration voltage of 20 kV; working distance depended on replica height but was typically between 8 and 12 mm. Replicas were examined in both 3D backscattered electron (BSE-3D) and backscattered electron topographic (BSE-TOPO) mode utilising Hitachi’s 5 segment backscatter detector. Typical cortical surface features with which fractures and other failure mechanisms interacted were examined and documented using areas of cortex peripheral to the cortical entry.

### Nomenclature for analysis

To accurately convey the orientation of structural features in the SEM images, the nomenclature presented in Fig. 1 is utilised for analysis. The *entry cortical fracture edge* (EnCF) meets the cortical surface at the *apex* (Fig. 1a, c), which defines the cortical entry when viewed from above. When viewed from below (Fig. 1b) and in cross section (Fig. 1c), the cortical entry is circumscribed by the EnCF. The tri-layered conoid formed by impact consists of a *cortical roof* and *cortical floor* with the trabeculae in between [4]. This conoid typically undergoes comminution but can leave behind traces in the form of cortical roof and floor fragments. When present, the *medial edge* of cortical roof fragment faces the EnCF, whilst the *distal edge* faces into the cortical entry.

## Results

### Cortical surface features

**Fig.3:**
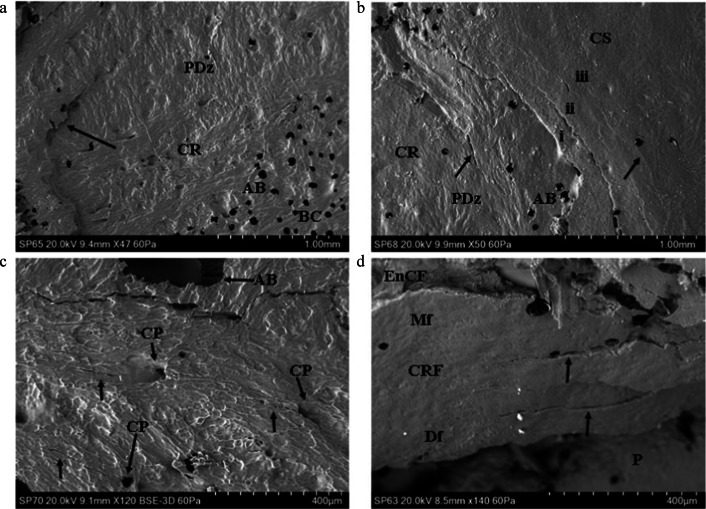
Genesis of the cortical entry in sandwich bones; plastic deformation and ring cracks (BC, base of compressed region; CS, cortical surface; CR, cortical roof; CRF, cortical roof fragment; Mf, medial fracture edge of cortical roof fragment; Df, distal fracture edge of cortical roof fragment; P, putty used to seal the floor of the entry; AB, air bubble artefact); **a** 54 m/s rebound impact, fleshed specimen (*Ei* 1.30 J; E´ 0.05 J/mm^2^), CS located just out of view top left and across the top of the micrograph; micrograph indicates extensive plastic deformation is involved in formation of the concavity (PDz); a ring crack is visible at left (arrow) with the base of the concavity located at the bottom right (BC); **b** 57 m/s rebound impact, fleshed specimen (*Ei* 1.44 J, E´ 0.05 J/mm^2^); micrograph of the right edge of a depressed fracture showing three generations of larger ring cracks (annotated i–iii) and two smaller cracks (arrowed); deformation forming the cavity occurs both along the ring crack fracture margins and in plastically deformed zones without visible fracture; **c** ~ 57 m/s impact (defleshed specimen) showing cortical surface adjacent to impact site (located along top of micrograph); concentrically orientated nascent ring cracks (white arrows) can be seen arising from cortical pores (CP); **d**, 96 m/s perforating impact (*Ei* 4.08 J, E´ 0.14 J/mm^2^); cortical roof fragment with the distal edge displaced downwards into the cortical entry and displaying concentric fractures consistent with ring cracking during the initial stages of impact (arrows)

The cortical surface of *Sus* scapulae presented varying degrees of *sculpturing* consisting of ridges combining to form a distinct tessellated pattern. This sculpturing varied from minimal (e.g., Fig. 3, a-b) to pronounced (e.g., Fig. 3c) across specimens. The cortex was punctuated intermittently by cortical pits (with a floor) and cortical pores (leading to a lumen), occasionally associated with a channel running along the cortical surface. Porosity varied across specimens, with some displaying few pores and some presenting with an abundance of them. The cortical surface showed no abrupt topographic change in slope from one region to another, and there were no distinct muscle attachment points in the outer cortical plate of any specimens.

### Casting artefacts; recognition and causation

Air bubble artefacts were introduced during the positive casting stage due to both the mixing process and exothermic curing. Small air bubbles, when present, were easily differentiated from cortical pores by their sharp edge and lack of internal morphology and did not influence feature identification (Fig. 3, a–b, AB). Larger air bubbles occasionally formed in portions of the cortical entry and, when present, prevented replication of surface detail and feature identification (Fig. 3c, AB). Comparison of micrographs to macrophotographs indicated that these larger bubbles may be associated with re-entrant geometries or large pores or depressions along the EnCF. Detail replication adjacent to these larger bubble artefacts was normal.

### Genesis of the cortical entry in sandwich bones

Rebound impact (impact velocities from 54 to 57 m/s) resulted in the formation of depressed discs of cortical bone, with previous work with µCT indicating that these form the cortical roof of tri-layered conoids if there is sufficient energy to drive fracture through all three sandwich bone laminae [4]. In all four rebound specimens, both plastic deformation and Hertzian fracture were operative under the impacting sphere, and both processes were responsible for formation of the concavity. Plastic deformation was often pronounced, with the plastically deformed zones descending steeply downwards to the base of the compressed region (Fig. 3, a–b). Plastic deformation occurred without formation of visible microcracks at the cortical surface, suggesting cracking was either too small to detect with the impression media or that an alternative failure mechanism is operative in the cortical bone.

Fracture in the compressed region in 3 of the 4 rebound impacts took the form of distinct circumferentially orientated *ring cracks* which occupied parts of the circumference, rather than completely encircling it (Fig. 3, a–b). Impact resulted in multiple generations of ring cracks extending from inside to outside of the cortical discs (Fig. 3b), with each ring crack formed successively at the widening contact radius of the projectile as it penetrated deeper into the cortex. In the fourth specimen, nascent concentric ring cracks less than 400 microns in length were observed emanating from cortical pores near the edge of the compressed region, indicating these had acted as stress concentrating flaws during impact (Fig. 3c). Separation of cortex at the ring cracks contributed to the observed compression of the cortical roof. Apparent ring cracks were also preserved on a section of cortical roof fragment resulting from a 96 m/s perforating impact (Fig. 3d, black arrows at right). Radial cracks arising normal to the apex of the EnCF were observed in one fleshed 54 m/s rebound impact (5 cracks) and one defleshed ~ 57 m/s impact (2 cracks). All were less than 500 µm in length apart from one large, approximately 1 mm crack in the cortex peripheral to the EnCF in the defleshed impact.

**Fig. 4:**
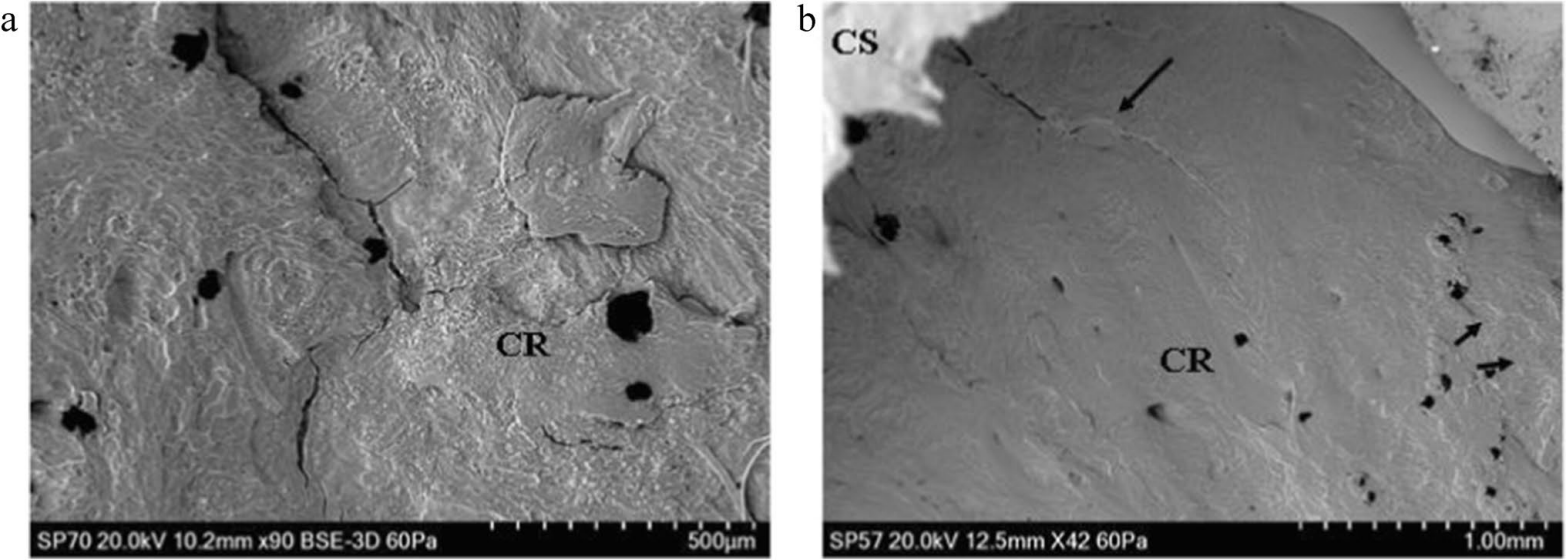
Genesis of the cortical entry in sandwich bones; failure of the cortical roof (CS, cortical surface; CR, cortical roof); **a** ~ 57 m/s rebound impact (defleshed specimen); surface of cortical roof showing vertically orientated fracture dividing the roof into two halves; fracture resulted from tensile failure of the inner surface of the cortical plate under compressive loading; **b** 75 m/s perforating impact (*Ei* = 2.47 J, E´ = 0.09 J/mm^2^), view of the cortical roof of a nearly intact conoid displaced downwards so it is beneath the cortical surface and protruding from the inner cortical plate; the surface has not been modified by impact but bears a fracture ending before the mid-section (arrow)

Initial failure of the cortical roof in both rebound and perforating impacts occurred via a fracture arising normal to the free fracture margin of the roof; this process was observed in both a rebound impact (Fig. 4a) and in the cortex of a relatively intact tri-layered conoid. In the latter case, the conoid had been displaced downwards and tilted so that the cortical roof was lower than the inner cortical layer (Fig. 4b). Despite this displacement, the surface sculpturing was not altered due to contact with the projectile. The same was true of the cortical surfaces of the two defleshed specimens subjected to rebound impact.

**Fig. 5:**
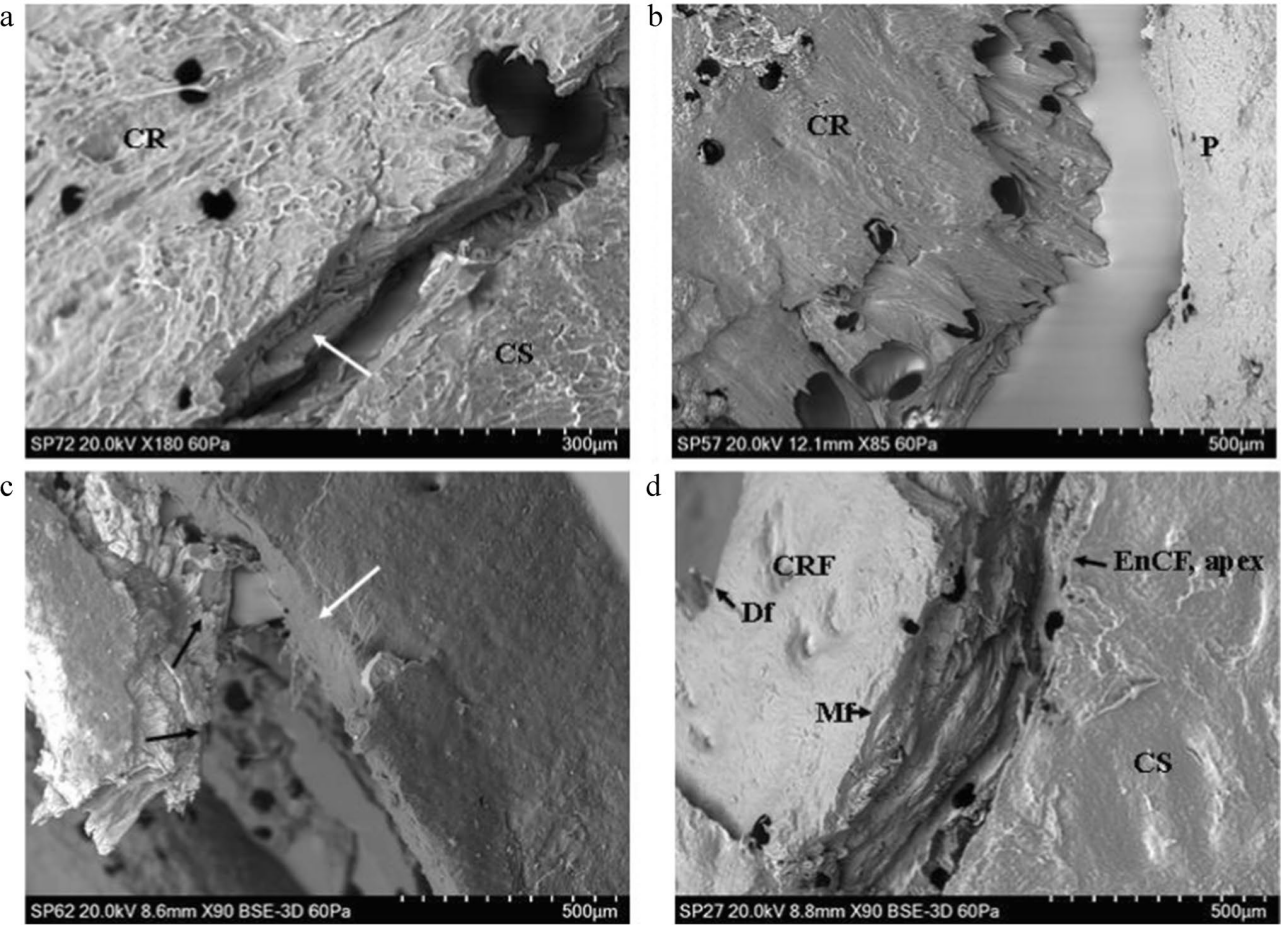
Genesis of the cortical entry in sandwich bones; stepping of the cortical roof(CS, cortical surface; CR, cortical roof; CRF, cortical roof fragment; Mf, medial fracture edge of cortical roof fragment; Df, distal fracture edge of cortical roof fragment; P, putty used to seal the cortical entry for negative moulding; EnCF, entry cortical fracture edge); **a** ~ 57 m/s rebound impact (defleshed specimen), mode I (tensile, crack opening) fracture mode indicated by stepping of the fracture edge across lamellar planes inside the cortical roof (arrow); **b** 75 m/s perforating impact (*Ei* = 2.47 J, E´ = 0.09 J/mm^2^) showing right fracture edge of the cortical roof of the nearly intact conoid seen in Fig. 4b, complex stepped morphology resulting from mode I fracture across lamellae; fracture edge is angulated to match the corresponding angulation of the EnCF; **c** 94 m/s perforating impact (*Ei* 3.94 J, E´ 0.14 J/mm^2^), cortical roof fragment with the distal surface facing upwards and displaying internal lamellae on the medial side orientated in different planes (highlighted by the small arrows); large arrow indicates an area of smooth bone consistent with being surface-modified plastic deformation; **d** 161 m/s perforating impact (*Ei* 11.44 J, E´ 0.40 J/mm^2^) showing cortical roof fragment with pronounced stepping across the lamellae of the medial fracture margin

Examination of the medial margin of both the cortical roof and cortical roof fragments in perforating impacts revealed some degree of stepping of the fracture edges resulting from mode I, tensile failure during cone crack propagation across the lamellae composing the fibrolamellar bone. This stepped morphology was observed on the margin of the cortical roof resulting from both rebound (Fig. 5a) and perforating impacts (Fig. 5, b–d). The stepped edges were either composed of pointed, overlapping lamellar components (Fig. 5, b–c) or presented a more uniform stepped morphology (Fig. 5d). The pointed lamellar morphology is illustrated in Fig. 5b, which shows the lower right margin of the cortical roof of the intact conoid seen in Fig. 4b. In this part of the conoid, the stepped region was angled to match the corresponding angulation of the EnCF, and the overlapping pointed lamellar fracture edges produced a complex fracture surface. This morphology may have resulted from the plane of the crack in relation to the lamellar structure in that region or from a pull-out process as the conoid was compressed downwards. Stepped fracture margins with pointed fracture edges were also observed on a smaller scale in a fragment of cortical roof resulting from a 94 m/s impact; in this specimen, the pointed ends of the lamellae revealed the differing orientations of individual lamellae in successive layers (Fig. 5c, black arrows).

### Plastic deformation of the cortex

**Fig. 6:**
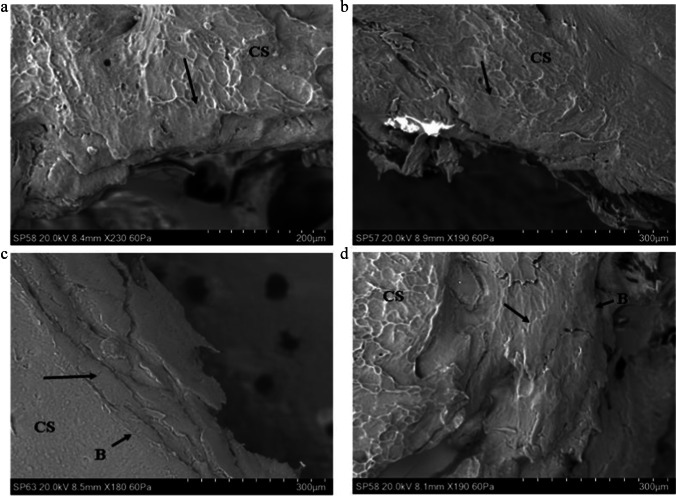
Surface-modified plastic deformation (SPD), 74–96 m/s perforating impacts (CS, cortical surface); **a** 74 m/s impact (*Ei* = 2.45 J, E´ = 0.09 J/mm^2^) showing SPD with transition of cortical sculpturing to smooth zone (black arrow); **b** 75 m/s impact (*Ei* = 2.47 J, E´ = 0.09 J/mm^2^) showing area of SPD with cortical sculpturing fading out at the point indicated by the arrow; bending results in a ductile morphology at the apex of the EnCF; **c** 96 m/s impact (*Ei* 4.08 J, E´ 0.14 J/mm^2^) with SPD showing compressed, deformed cortex (top black arrow) and bending without fracture (B) leading to a ductile morphology; **d** same specimen as in **a** showing SPD on a descending fragment of cortical roof, arrow indicates the point at which cortical sculpturing fades; bending at **B** is extensive enough to allow the fragment to descend into the cortical entry without fracture

**Fig. 7:**
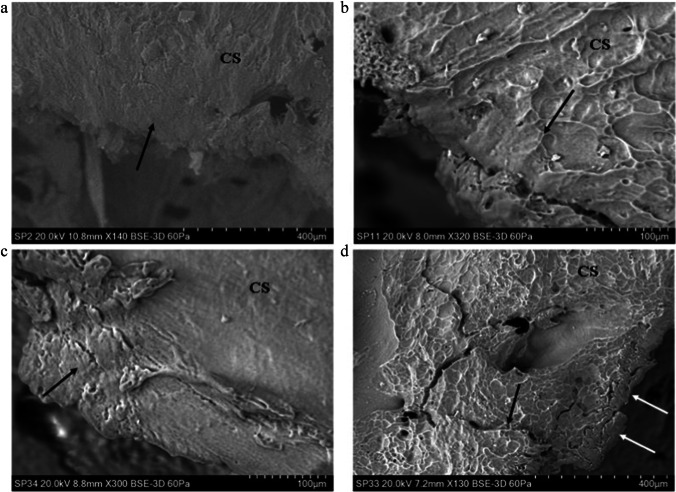
Surface modified plastic deformation (SPD), 149–609 m/s perforating impacts (CS, cortical surface); **a** 149 m/s impact (*Ei* = 9.78 J, E´ 0.35 J/mm^2^) showing loss of surface detail (arrow); **b** 333 m/s impact (*Ei* = 49.07 J, E´ = 1.74 J/mm^2^) showing SPD with transition of cortical sculpturing (arrow), **c** 591 m/s impact (*Ei* = 154.37 J, E´ = 5.46 J/mm^2^) showing SPD (black arrow) with small transverse fractures; **d** 609 m/s impact (*Ei* = 163.89 J, E´ = 5.80 J/mm^2^) showing SPD along the apex of the EnCF (lower white arrows) in relation to the immediate cortical landscape; note the radial crack emanating from the EnCF (black arrow)

**Fig. 8:**
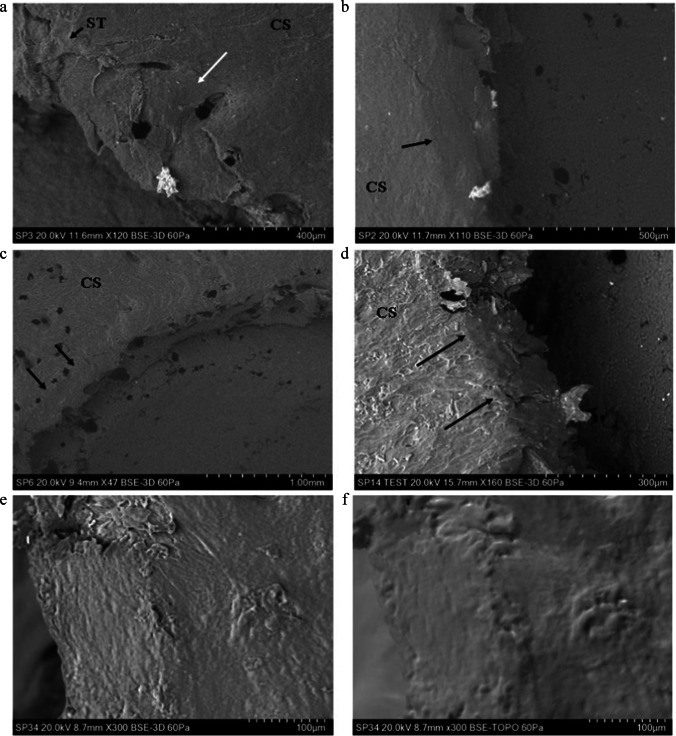
Peripheral plastic deformation and associated ductile morphology (perforating impacts), (CS cortical surface; ST, soft tissue); **a** 163 m/s impact (*Ei* = 11.72 J, E´ = 0.41 J/mm^2^), showing small area of plastic deformation with intact surface sculpturing; note that the CS is continuous with the bent section of bone (white arrow); **b** 149 m/s impact (*Ei* = 9.78 J, E´ 0.35 J/mm^2^), area of plastically deformed bone with intact surface sculpturing and fold identified by arrow; this area is at the lower edge of a section of cortical roof fragment; **c** 165 m/s impact (*Ei* = 12.05 J, E´ = 0.43 J/mm^2^) showing margin of cortical entry with cortical bending due to plastic deformation, particularly at the area identified by black arrows; **d** 570 m/s impact (*Ei* = 143.96 J, E´ = 5.09 J/mm^2^); area of plastic deformation with the fold identified by arrows; note that this region combines pronounced cortical sloping with some surface modification; **e** 591 m/s impact (*Ei* = 154.37 J, E´ = 5.46 J/mm^2^) showing figure continuity of the cortical surface with the plastically deformed region; **f** topographic view of **e **confirming bending of the cortex

In addition to the plastic deformation seen in the rebound impacts, analysis of the apex of the EnCF and adjacent cortical surface revealed that the cortex underwent plastic deformation in perforating impacts, including two at over 850 m/s. Plastic deformation in this region occurred in two distinct forms, which were identifiable based upon morphology and location. *Surface-modified plastic deformation (SPD)* (Figs. 6–7) was characterised by loss of surface sculpturing and varying extents of downward deformation (bending) without visible fracture. SPD was entirely restricted to the *projectile-bone interface*, being found only at the apex of the EnCF and the surfaces of cortical roof fragments inside the cortical entry. *Peripheral plastic deformation* involved the cortical surface peripheral to the apex, with the cortical surface typically remaining unmodified or partially modified (Fig. 8). These different types of plastic deformation are discussed below.

### Surface-modified plastic deformation

In total, SPD was identified in 18 of the 21 perforated specimens spanning a velocity range from 73 to 871 m/s. SPD, which was localised around the apex rather than occupying the full circumference, was characterised by the gradual loss of surface sculpturing at the apex of the EnCF with an eventual *transition zone* where the ridges blended with smooth areas of cortical bone at the projectile-bone interface. This transition zone is illustrated in specimens impacted at 74–75 m/s Fig. 6, a–b and in specimens impacted at 149–333 m/s in Fig. 7, a–b. Additional features of SPD, present to varying extents in each affected region, were a compressed, deformed cortex and down-bending in the absence of visible fracture. These features are illustrated in a 96 m/s impact in Fig. 6c and in a 591 m/s and 609 m/s impact in Fig. 7 c–d, respectively. The down-bending associated with SPD resulted in a ductile morphology at the apex of the EnCF. Figure 6d shows a cortical roof fragment where bending was extensive enough to allow it to descend into the cortical entry without visible fracture at the top edge (B in the image). Areas of SPD sometimes showed circumferential fractures running along the modified cortical surface (Fig. 7c); these were indistinguishable from ring cracks when they were of large size. The contrast between the cortical surface in the immediate cortical landscape and SPD along the apex is illustrated in Fig. 7d. In this example, the cortical surface adjacent to the apex was markedly sculpted; this morphology contrasted greatly with the region of SPD which showed complete loss of sculpturing, bending without visible fracture and a compressed, smoothed appearance.

### Peripheral plastic deformation (PPD)

PPD was detected in 7 of the 21 perforated specimens with an impact velocity range of 73 to 591 m/s. In common with SPD, PPD did not occupy the full circumference of the cortical entry and was localised in distribution. PPD was characterised by down-bending of the cortex a variable distance peripheral to the apex of the EnCF with little to no alteration to cortical sculpturing. As a result of this process, the apex was characteristically lower than the cortical surface. The sloping associated with PPD imparted a ductile morphology to the periphery of the cortical entries in affected regions (Fig. 8). PPD was identified at the attached ends of cortical roof fragments (Fig. 8, a–b) and diffusely around the margins of the apex (Fig. 8, c–e). The slope induced by plastic deformation varied from being gradual (Fig. 8a) to steep, and in the latter case, the area where bending initiated could be easily identified as a distinct fold (Fig. 8, b–f). The ductile morphology induced by PPD when it occupied a larger portion of the circumference of the cortical entry is shown in Fig. 8c, where the bending at left is approximately 1-mm long. Figure 8d shows an image of a specimen impacted at 570 m/s, which exhibited a very steep deformed region. In this case, the fold was well-defined, and the slope steep enough for the apex of the EnCF to nearly contact the putty which was situated lower than the outer cortical plate (located at the right of the image). Continuity of the cortical surface to a deformed region is shown in Fig. 8e, which shows a small area of PPD from above and at × 300 magnification in a specimen impacted at 591 m/s. Topographic view of the same region highlights both the extent of sloping and the continuity of the cortical surface across it (Fig. 8f).

### Cortical morphology when SPD and PPD were absent

**Fig.9:**
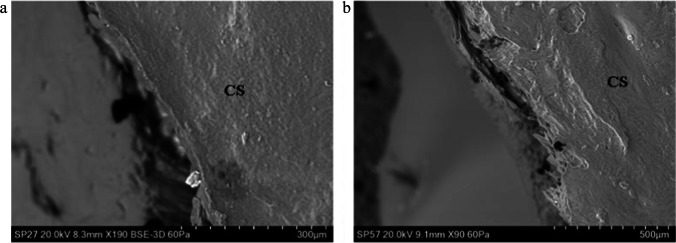
Apex of the EnCF in non-plastically deformed zones, perforating impacts (CS, cortical surface); **a**, 161 m/s impact (*Ei* 11.44 J, E´ 0.40 J/mm^2^), abrupt and straight-edged apex; **b**, 75 m/s impact (*Ei* = 2.47 J, E´ = 0.09 J/mm^2^), showing more complex fracture morphology with re-entrant geometries; both fracture edges seen here differ markedly from zones of SPD and PPD

Neither SPD nor PPD were distributed uniformly around the whole circumference of the cortical entries and when absent the EnCF presented with abrupt edges (Fig. 9a) or complex edges with re-entrant geometries between fragmented cortical components (Fig. 9b).

### Radial cracks; perforating impacts

**Fig. 10:**
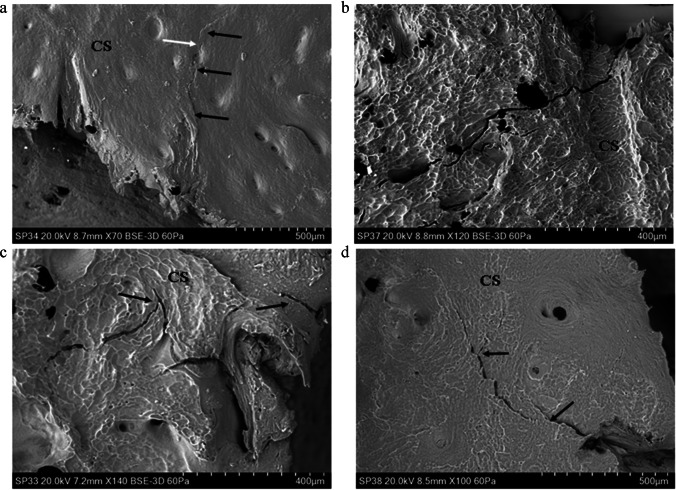
Radial cracks originating from the entry cortical fracture edge, perforating impacts (CS, cortical surface)**; a** 591 m/s impact (*Ei* = 154.37 J, E´ = 5.46 J/mm^2^), narrow crack crossing cortical pit and showing a small ligament bridge (white arrow); **b** 850 m/s impact (*Ei* = 319.60 J, E´ = 11.31 J/mm^2^) showing large, wide radial crack crossing cortical furrow; **c** 609 m/s impact (*Ei* = 163.89 J, E´ = 5.80 J/mm^2^), showing crack with a bifurcation (left arrow) and a separate straight radial crack inside a cortical furrow (right arrow); **d** 871 m/s impact (*Ei* = 335.71 J, E´ = 11.88 J/mm^2^), long radial crack with two points of bifurcation (arrows)

Radial cracks were observed in 19 of the 21 perforated specimens across the velocity series (illustrated for impacts from 591 to 871 m/s in Fig. 10, a–d). Radial cracks were identified in 12 of the 14 impacts between 58 and 168 m/s and in all 7 impacts between 333 and 897 m/s. Radial cracks followed both relatively straight (Fig. 10, a–b) and tortuous (Fig. 10c) paths. Tortuosity is not clearly associated with velocity; for example, the tortuous crack in Fig. 10c is associated with a 609 m/s impact, whilst the straight crack in Fig. 10b is created by an 850 m/s impact. Some of the radial cracks exhibited uncracked ligament bridging despite high impact velocity (e.g., white arrow in Fig. 10a). Radial cracks ranged from being narrow (Fig. 10a) to open (Fig. 10, b–d) and occasionally exhibited bifurcations along their path (Fig. 10, c–d, arrows). Estimates of crack length using the scale in the images indicated that radial cracks > 1 mm in length were more common in high-velocity impacts. Of the 14 perforated specimens impacted between 58 and 168 m/s, only three possessed radial cracks longer than 1 mm (impacted at 58 m/s, 165 m/s and 167 m/s, respectively). In contrast, a total of 4 out of 6 specimens impacted between 333 and 897 m/s had radial cracks greater than 1 mm in length (impacted at 591 m/s, 850 m/s, 885 m/s and 897 m/s).

### Circumferential cracks and cracks arising from cortical stress concentrators

**Fig. 11:**
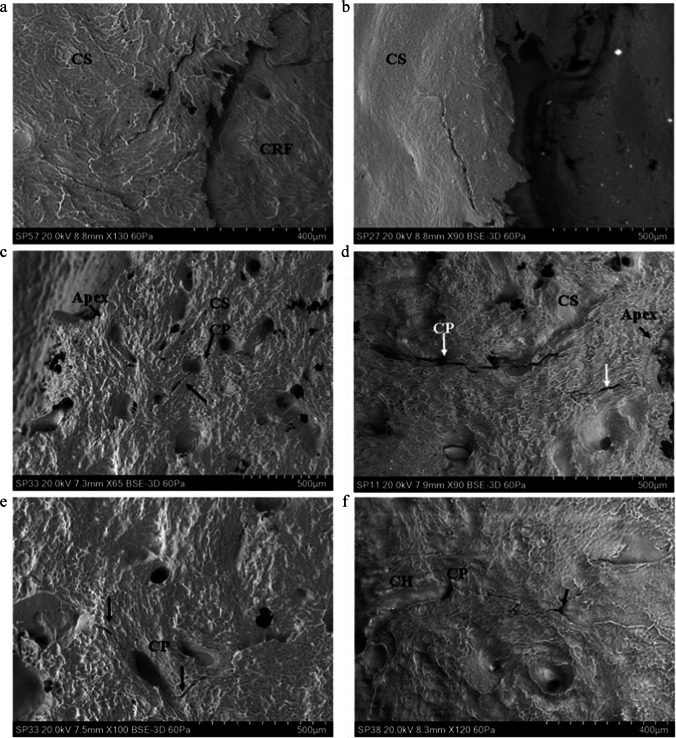
Concentric cracking and cracks arising from cortical stress concentrators, perforating impacts (CS, cortical surface, CR, cortical roof; CRF, cortical roof fragment, CP, cortical pore; CH, channel); **a** 75 m/s impact (*Ei* = 2.47 J, E´ = 0.09 J/mm^2^), showing concentric crack adjacent to a fragment of cortical roof; **b** 161 m/s impact (*Ei* 11.44 J, E´ 0.40 J/mm^2^) with concentrically orientated crack near the EnCF; **c** 609 m/s impact (*Ei* = 154.37 J, E´ = 5.46 J/mm^2^) showing concentric crack arising from cortical pit adjacent to the apex of the EnCF; **d** 333 m/s perforating impact (*Ei* = 49.07 J, E´ = 1.74 J/mm^2^), large radially orientated crack adjacent to the apex and associated with a cortical pore; note the smaller radially orientated crack (lower arrow) that arises within the cortex not a pore; **e** same specimen as in **c**, showing elliptical cortical pore with the major axis running upwards from right to left; stresses acting normal to this axis resulted in stress concentrating at the tips of the elliptical cavity and resulted in cracking at both ends [34]; **f**, 871 m/s perforating impact (*Ei* = 335.71 J, E´ = 11.88 J/mm^2^) showing figure crack associated with a cortical pore/ channel complex and extending to a smaller pore-type structure at right (arrow)

Circumferential cracks were identified in a total of 9/14 impacts between 58 and 168 m/s and 4/7 impacts between 333 and 897 m/s. These cracks were found both near the apex of the EnCF (Fig. 11, a–b) and further away in the cortical landscape; in one case, a concentric crack was found approximately 1.5 mm from the cortical entry. Unlike the rebound impacts, circumferential cracks in the perforating impacts did not appear to form concentric generations but were dispersed diffusely around the cortical entry. Cortical pits and pores acted as stress concentrators during impact and were detected as the origin for some circumferential (Fig. 11c) and radial (Fig. 11d) cracks. In elliptical pores, stresses acting normal to the major axis resulted in cracking at each end due to concentration of stresses there (Fig. 11e) [34]. Pore and pit cracks did not always occur in isolation, with an example of a linked pore-crack complex occurring in an 871 m/s impact (Fig. 11f). Cortical pore cracking occurs both near the apex of the EnCF (Fig. 11, a–b) and more distant, with the pore in Fig. 11e being over 1 mm away from it.

## Discussion

A central tenet of the present paper was that the process of projectile trauma analysis and diagnosis will be enhanced when the penetration mechanisms and their associated morphological characteristics are defined at multiple hierarchical scales of bone organisation. SEM analysis of perforating impacts from 58 to 897 m/s revealed that at the microscopic scale, the cortical entry and surrounding cortical surface are characterised by varying degrees of SPD and PPD, by radial cracks emanating at or near to the apex, by circumferential cracks running parallel to the apex, and by pore/pit-crack complexes up to 1.5 mm from the apex. Areas of apex lacking plastic deformation were either abrupt or exhibited complex re-entrant geometries. Evidence for tensile, mode I failure due to impact consisted of stepped fracture edges on the medial edge of intact cortical roofs in rebound impacts and cortical roof fragments in perforating impacts. It is interesting to note that peripheral plastic deformation, radial cracks, and concentric cracks have been identified around a perforating fracture to a human parietal [6], indicating that at least three of the features observed here also occur in human compact bone. Although further work is required to establish the prevalence of these features in human bone, their potential as diagnostic indicators is readily apparent. For example, whilst GSR cannot be utilised as diagnostic evidence when environmental contamination cannot be ruled out [15], the presence of GSR with a combination of the above microscopic features might substantiate projectile involvement. Similarly, these features may be of use when only a small fragment of a perforating hole is recovered or when differentially diagnosing intact holes of ambiguous aetiology.

In concurrence with previous evidence that conoidal projectile wounds in sandwich bones are created by cone cracking [3, 4], SEM analysis of rebound impacts in the present study confirmed the formation of ring cracks in compact bone impacted by spherical projectiles. Ring cracks are shallow, circular cracks that form normal to the surface at the edge of the impactor contact radius which, at a certain load (*P*), then propagate into the material whilst flaring outwards to form a cone crack [35]. Ring cracks themselves originate from surface flaws activated by the high tensile stresses present at the edge of the contact circle [36]. Although the flaws giving rise to the ring cracks in this study were too small to be identified in three cases, in one rebound impact nascent concentric ring cracks were observed originating from cortical pits and pores adjacent to the apex. Ring cracks in cortical bone are identical in form to those produced by indentation or impact in other ceramics [35, 37] and thus emphasise the importance of the mineral phase of bone in determining the dominant penetration mechanism.

Analysis of the impact site across specimens indicated that there were differences in the number of generations of ring cracks formed during penetration. In three rebound impacts, several generations of ring cracks were formed as the projectile penetrated progressively deeper into the cortex. Fractures consistent with being two generations of ring cracks were also preserved in a cortical roof fragment resulting from a 96 m/s impact. In contrast, SEM analysis of the intact cortical roof of the conoid produced at 74 m/s did not show any ring cracks (see [4] for µCT cross-sectional analysis of this conoid). Ring cracks large enough to detect with µCT were also absent from a second conoid resulting from a 57 m/s impact in the same experimental series [4] and are not present in the images of intact conoids reported in the literature [38–40]. Variation in ring crack number and distribution around the impact site has also been demonstrated in other ceramic materials including dense alumina [35] and coarse alumina subjected to repeated load cycles [37].

In bone conoids, absence of multiple generations of ring cracks may be explained by compression brought about by plastic deformation and fracture of the cortical roof, both of which have been observed in cross section [4]. Plastic deformation can occur in brittle materials during indentation [40], with experimental work in ceramics indicating that inelastic deformation occurs before fracture if the projectile radius is below a critical value [41]. Theoretically, compression due to plastic deformation and/or fracture would cause immediate contact with a wider projectile radius and thus result in only one ring crack. In support of this hypothesis, four of the five conoids described from the archaeological and forensic literature displayed a compressed cortical roof [38–40], as did the two most intact conoids in our previous experimental series [4]. This hypothesis cannot fully explain lack of multiple ring cracks, however, as the rebound impacts exhibited both plastic deformation and more than one generation of ring crack. In addition to compression, it is therefore likely that bone parameters such as cortical thickness, mineralisation and geometry at the site of impact, in addition to the angle of incidence of the projectile in relation to the bone, may be involved in determining the ring cracking behaviour of the cortical roof.

In addition to plastic deformation of the cortical roof in rebound impacts, plastic deformation in the form PPD and SPD was observed in perforating impacts induced from 74 to 971 m/s. Whilst permanent deformation identical in form to PPD has been previously been observed in bovine scapulae impacted with a captive bolt and 7.62 × 51 mm NATO full metal jacket projectiles [6], to the best of our knowledge, this is the first study to report bending associated with loss of surface sculpting in the form of SPD. These plastic deformation morphotypes differed both in their location and appearance. Whilst SPD was restricted to the projectile-bone interface at the apex, PPD started in the cortex adjacent to it and where present imparted a distinct dish-shaped morphology to the cortical entry. Interestingly, no cortical entries displayed SPD or PPD around their entire circumference; accordingly, a given cortical entry might express areas of plastic deformation that contrast sharply with abrupt, non-deformed edges.

The presence of plastic deformation of any kind around fractures induced at higher velocities is surprising and suggests involvement of different plastic mechanisms to those operative at lower loading rates. In material terms, bone is a semi-brittle solid whose viscoelastic properties impart strain rate sensitivity to its failure behaviour [34, 42]. Thus, whilst low strain rates allow bone to deform in the plastic part of the stress–strain curve, high strain rates apply energy too quickly for the viscous mechanisms to respond and the bone fractures in brittle fashion [2, 32, 43, 44]. Accordingly, mechanisms that allow bone to deform in the plastic portion of the curve, including slip within and between mineralised collagen fibrils [45–48] and microcracking [49], cannot account for plastic deformation during impulsive loading. The questions therefore arise as to how plastic deformation is occurring between 74 and 871 m/s and if the mechanisms are the same for SPD and PPD.

One possible underlying mechanism for the observed ductility that has some experimental support is softening of the mineral phase in the cortical bone surrounding the impact site. Previous analysis utilising nanohardness measurements of cortical entries in human sandwich bones has indicated a loss of hardness from 61.1 ± 1.6 kg/mm^2^ in a control specimen to 47.7 ± 0.9 kg/mm^2^ in cortical bone adjacent to the entry [30]. These authors attributed the softening to shock-induced phase changes and resultant amorphization of hydroxyapatite (HA) crystals. The latter process was also considered to cause an increase in the size of nanopores around the collagen fibres, disrupting their organisation and resulting in increased porosity of the bone matrix. Reversible pressure-induced structural change has been demonstrated experimentally in pure HA crystals at 20 kilobars (kbar), with reversal occurring during decompression at a pressure of 22 kbar [50]. Later analysis of HA with X-ray diffraction (XRD), Fourier transform infrared (FTIR) and SEM has demonstrated a transition from crystalline to fully amorphous phase at 10 Gigapascals (GPa) or 100 kbar pressure [51]. The effect of shock waves may also be more apparent in bone than other tissues due to its density, which results in high amplitude reflections [27].

Whilst softening of the mineral phase may account for the ductility observed in the present study, any proposed mechanism must also account for the fact that plastic deformation was localised around the cortical entry. It is established that ceramics can undergo plastic deformation during shock loading through a process termed microplasticity, which in polycrystalline ceramics occurs due to deformation within and between crystals [52]. Experimental modelling of this process has demonstrated that microplasticity originates in localised sites that coalesce with an increase in strain, with subsequent microplastic evolution being determined by crystal orientation with respect to the applied load [52]. Significantly, this model also predicted that 2% of the ceramic must be undergoing microplasticity for plastic deformation to be observed macroscopically [52]. Although this model was not based on a nanocrystalline ceramic such as bone, a similar sensitivity to crystal orientation may explain the localised plastic deformation around the cortical entry, with collagen fibre and thus crystal orientation varying between bone lamellae around the circumference of the EnCF. Direction-specific behaviour such as this is consistent with the anisotropic nature of bone as a material [34]. Shock waves initiated by high-velocity impact also reflect at interfaces and cavities within the bone, a process that results in waves both reinforcing and cancelling cancel each other out [34]. The highly heterogeneous nature of bone and resultant complexity in shock wave behaviour around the impact site would also contribute to the localisation process. Further systematic analysis of the crystal structure in deformed and non-deformed regions of the cortical entry is necessary to confirm the distribution of shocked HA crystals in each region, and additional nanohardness investigations might also prove instructive.

Until the precise mechanisms underlying these plastic deformation types are elucidated, it is only possible to speculate as to whether they are formed in the same way. However, restriction of areas of SPD to the projectile-bone interface, that is, at the apex and on cortical roof fragments, is suggestive of it being caused by *direct close interaction* with the projectile. SPD was characterised by gradual loss of surface sculpturing resulting in smooth regions of bone, often with compressed appearance, and by bending of affected regions resulting in a ductile morphology. Similar areas of smooth bone at the EnCF have been observed in a previous SEM analysis of porcine ribs impacted with 0.22 bullets and were attributed to melting of the bone due to frictional heating [31]. However, although a portion of the available incident kinetic energy will be turned into frictional heat, it is not currently known what temperatures might be generated during perforation of cortical bone or if the contact time is sufficient to allow heat to transfer. Although data is lacking for bone, published data for synthetic materials provides some insight into the temperatures that can be created by impact. Thermographic analysis of the penetration of ultra-high molecular weight polyethylene with fragment simulated projectiles captured a temperature at the site of impact of 200 °C, with thermal energy accounting for half of the lost incident kinetic energy during perforation [53].

The effects of heat on bone chemistry are complex, but what is clear is that temperatures must be high and sustained to bring about change to the crystal structure. Experimental work has shown that bone morphology remains normal up to 185 °C and that melting of HA only occurs at temperatures exceeding 800 °C [54]. Furthermore, a change in HA crystal structure has been reported to occur only in the first 15 min of heating at 500 °C [55]. Combined, this data suggests that the smooth areas of bone reported previously [31] and categorised as SPD in the present study are unlikely to result from melting of the hydroxyapatite. As an alternative hypothesis, it is speculated that the observed morphology of SPD is the result of shock-induced softening coupled with a physical scraping and crushing effect at the projectile-bone interface. Maximum stress wave intensity at the apex of the EnCF and extreme physical disruption of the HA crystals may account for the gradual loss of cortical sculpturing at the apex, with alteration in crystal structure at the nanoscale leading to morphological change at the microscale.

Apart from one specimen impacted at 885 m/s that exhibited two macroscopic radial fractures, impacted porcine scapulae did not show the large radial or concentric fractures that are commonly associated with gunshot entry and exit sites in human crania. These fracture types are also typically absent in bovine scapulae subjected to ballistic impact [6] but have been documented in bovine ribs [56]. Radially and concentrically orientated cracks were identified around the cortical entry at the microscopic scale in both re-bound and perforating impacts, but whilst the radial cracks may be analogous to those seen macroscopically in human material the concentric cracks are not. Concentric cracks associated with ballistic trauma in human crania originate under tension in the endocranium as a result of temporary cavitation within the brain [57], whilst the microscopic fractures originated in the outer cortical plate in the absence of cavitational effects. Although further quantitative work is required, preliminary analysis indicated that radial cracks over 1 mm in length were more common with higher velocity impacts. An increase in crack length such as this is consistent with the greater absolute amounts of kinetic energy absorbed at higher impact velocities, which allows greater work of fracture. Uncracked ligament bridging was observed in cortical cracks from 54 m/s up to the highest impact velocity of 897 m/s. This phenomenon, which occurs when a secondary crack initiates slightly ahead of a primary crack with a segment of cortex between the two, is a well-documented toughening mechanism in bone [48]; however, this appears to be first time it has been observed adjacent to projectile perforation sites.

The microscopic radial cracks observed in the present study may have some taphonomic significance. Calcination of cortical entries in bovine ribs by heating at 800 °C resulted in the nucleation of large radial cracks from the apex that were not there before heating [56]. Heating drill holes in the same bone type and subjecting them to the same temperature did not result in radial cracking, and as a result, the authors speculated that injury-related fractures, such as ballistic impact, induced areas of weakness which they termed *loci minoris resistentiae* [56]. Although they did not speculate as to what the cause of this weakness might be, thermal expansion of microscopic radial cracks emanating at or near the EnCF would explain the additional macroscopic radial cracks during heating.

Whilst previous cross-sectional analysis with µCT has demonstrated damage localisation through the depth of the conoidal fracture [3], finite element modelling of von Mises stresses has demonstrated that the stress field does radiate some distance from the cortical entry [58–62]. SEM analysis in the present study indicates that this stress field is sufficient to nucleate cracks from cortical pits and pores in the immediate cortical environment around the cortical entry. Crack nucleation from these cortical features followed predictable physical laws, with cracks emanating from the ends of ellipsoidal pores or pits where the stress concentration is highest [34]. Further quantitative analysis controlling for pit/pore density should seek to address if the abundance of these small cracks around a given cortical entry is related to the magnitude of the stress field.

## Conclusion

The present paper sought to begin addressing the question as to whether there is a level of bone organisation where the energy inputs and contact stresses associated with high-velocity projectile trauma might manifest structurally. In this regard, the presence of plastic deformation and the indication that this is associated with a structural change at the level of individual HA crystals are significant. Plastic deformation was induced by both low- and high-velocity impacts, and therefore, future analyses should seek to characterise the precise changes to crystal structure and/or orientation that are occurring both in PPD and SPD and then establish if there is a quantitative relationship between the degree of structural change and absorbed kinetic energy during the impact event. Further analyses of radial crack length and tortuosity are also necessary. Resolution with the negative–positive casting process was in the order of microns, confirming the utility of this process in the examination of traumatic fractures. Further work should seek to develop techniques to reduce artefact formation and allow enhanced quantitative analyses.

This experimental study utilised a simple projectile-target combination to provide baseline data on the morphological features associated with projectile impact and penetration. Future studies should seek to sequentially introduce additional projectile design variables, such as variation in tip shape, to determine any associated morphological effects. Whilst larger sample sizes, ease of acquisition and storage are of benefit with the animal model utilised here, further work with human material from both case and experimental series is required to document the microstructural characteristics of the cortical entry in human cortical bone. In sum, the findings of the present work indicate that a comprehensive understanding of projectile trauma must include analysis across lower scales of organisation. Differential diagnosis will be enhanced when the failure mechanisms, and associated morphological characteristics are defined and documented across multiple hierarchical scales.

## Declarations

### Compliance with involving human and/or animals

This study was fully authorised by the ethics committee of Cranfield University. Porcine scapulae were obtained from animals humanely killed as part of the food chain in full accordance with strict UK animal welfare standards.
